# SOCIAL CAPITAL AND THE PET EFFECT

**DOI:** 10.1093/geroni/igz038.1948

**Published:** 2019-11-08

**Authors:** Judith Robertson R Phillips, Edith Jimenez, Heather Nicholson

**Affiliations:** California State University San Marcos, San Marcos, California, United States

## Abstract

Social capital such as positive relationships and social support play an important role in older adults’ well-being. Typically, researchers have investigated family and friends as providers of interpersonal resources to older adults but there has been an increasing trend to explore companion pets as providers of social capital and to investigate the impact of pet companionship on older adults’ psychological well-being. Inconsistencies have appeared in the literature though as to whether there is a “pet effect,” the positive benefit of companion pets on older adults’ psychological health. The purpose of this study was to investigate whether having a companion pet would provide greater social connection and better psychological well-being among 83 community-residing older adults (meanage = 62.87 years; males = 38; females = 45) who owned a companion pet, a dog (n= 53) , cat, (n= 21) or both (n= 9), versus 42 community-residing older adults (meanage = 65.69 years; males = 25; females = 17) who didn’t own companion pets. Analyses revealed that no “pet effect” was found for any measure of psychological well-being: self-reported loneliness, happiness, life satisfaction, or mental health. This was especially true for cat owners in that the more one viewed a cat as a family member, the lower one’s life satisfaction and happiness. In addition, as the number of cats in the household increased, the perceived social support from a significant other, family, and friends lowered. Discussion will focus on the implications of these results for pets as providers of social capital.

